# Microelectromechanically tunable multiband metamaterial with preserved isotropy

**DOI:** 10.1038/srep11678

**Published:** 2015-06-26

**Authors:** Prakash Pitchappa, Chong Pei Ho, You Qian, Lokesh Dhakar, Navab Singh, Chengkuo Lee

**Affiliations:** 1 Department of Electrical and Computer Engineering, National University of Singapore, 4 Engineering Drive 3, Singapore 117576; 2 Center for Intelligent Sensors and MEMS, National University of Singapore, 4 Engineering Drive 3, Singapore 117576; 3 Institute of Microelectronics, A*STAR (Agency for Science, Technology and Research), 11 Science Park Road, Singapore Science Park II, Singapore 117685.; 4 NUS Suzhou Research Institute (NUSRI), Suzhou Industrial Park, Suzhou, P. R. China 215123

## Abstract

We experimentally demonstrate a micromachined reconfigurable metamaterial with polarization independent characteristics for multiple resonances in terahertz spectral region. The metamaterial unit cell consists of eight out-of-plane deformable microcantilevers placed at each corner of an octagon ring. The octagon shaped unit cell geometry provides the desired rotational symmetry, while the out-of-plane movable cantilevers preserves the symmetry at different configurations of the metamaterial. The metamaterial is shown to provide polarization independent response for both electrical inductive-capacitive (eLC) resonance and dipolar resonance at all states of actuation. The proposed metamaterial has a switching range of 0.16 THz and 0.37 THz and a transmission intensity change of more than 0.2 and 0.7 for the eLC and dipolar resonances, respectively for both TE and TM modes. Further optimization of the metal layer thickness, provides an improvement of up to 80% modulation at 0.57 THz. The simultaneously tunable dual band isotropic metamaterial will enable the realization of high performance electro-optic devices that would facilitate numerous terahertz applications such as compressive terahertz imaging, miniaturized terahertz spectroscopy and next generation high speed wireless communication possible in the near future.

Electromagnetic metamaterial (EM) is an array of subwavelength structures that can be engineered to achieve exotic material properties such as artificial magnetism^1^, negative refractive index^2^, unnaturally large refractive index^3^, chirality^4^, phase engineering^5^, beam steering^6^, polarization conversion^7^, etc. in desired spectral region of interest. More interestingly, these devices enable flat devices that are very attractive from system-level application perspective^5^,^6^,^7^. The EM properties of the metamaterials can be dynamically altered through external stimulus and are known as “active metamaterials”. Two popular approaches have been reported for the realization of active metamaterials– a) changing the material property of either a part of metamaterial unit cell or the surrounding medium of the metamaterial and b) through physical reconfiguration of metamaterial unit cell using microactuators. Conventional tuning method of changing the material properties requires active materials to be a part of metamaterial, which can then be controlled through one of various possible external signaling methods such as optical^8^,^9^,^10^, electrical^11^,^12^,^13^,^14^,^15^,^16^,^17^, magnetic^18^,^19^ and thermal^20^,^21^ inputs. The semiconductor based actively tunable metamaterial through photo-excitation^8^,^9^,^10^ and electrical bias^11^,^12^,^17^ provides high modulation depth with faster response time. However, the thermally^20^,^21^ or magneto- statically^18^,^19^ tunable metamaterial have slower response time or demand bulky setup to provide active controls and hence are not very attractive as modulators. The lack of active materials with the desired properties at terahertz (THz) frequencies greatly limits the scaling of device dimensions to operate efficiently in the THz spectral region. Furthermore, active tuning of LC mode resonance is reported using these conventional methods, and so are polarization sensitive. Few reports on dipolar resonance tuning has been reported with polarization insensitive characteristics but with limited electro-optic response^22^,^23^,^24^. On the other-hand, microelectromechnical systems (MEMS) based reconfigurable metamaterials (MRM) with in-plane actuation shows a continuously varying anisotropy^25^,^26^,^27^,^28^,^29^,^30^, while for out-of-plane movable actuator based metamaterial lacks the rotational symmetry of the unit cell, due to the routing metal lines^31^,^32^,^33^,^34^,^35^,^36^,^37^,^38^,^39^,^40^. It is of great importance to realize high performance, polarization independent, active metamaterials for a wide range of impactful THz applications such as spectroscopy, ellipsometry and imaging.

## Uniaxially isotropic tunable MEMS metamaterial structure

**Figure 1:**
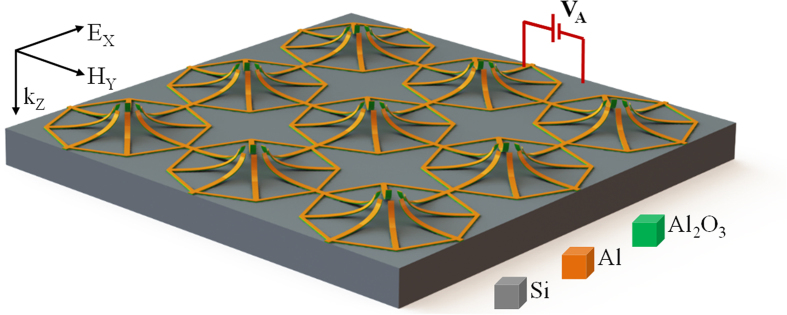
Illustrative schematics of the proposed uniaxially isotropic MEMS metamaterial with eight released cantilevers in an octagon ring.

**Figure 2:**
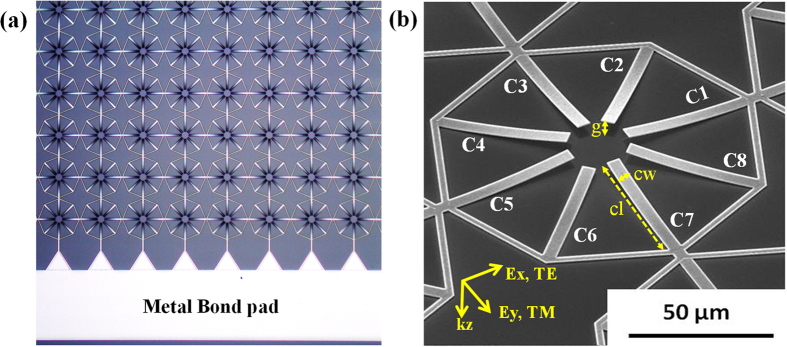
(**a**) Optical microscope image of the fabricated MRM array and (**b**) Scanning electron microscope image of fabricated unit cell of metamaterial in OFF state with the geometrical parameters definitions.

In this report, we demonstrate a MEMS tunable dual band metamaterial with polarization independent characteristics for TE and TM incidence mode in THz spectral region. In order to achieve polarization independent characteristics in all states of reconfiguration, the unit cell pattern should have rotational symmetry in the plane perpendicular to the wave propagation direction. Also, the actuation direction should be along the direction of incident wave propagation (i.e. perpendicular to the plane containing the electric and magnetic field of the incident wave). The proposed MRM unit cell consists of eight geometrically identical microcantilevers (C1–C8) placed symmetrically at eight corners of an octagon ring as shown in Fig. 1. The octagon rings in adjacent unit cells are electrically connected to each other, thereby forming the routing lines to provide electrical input to all suspended cantilevers. Owing to the rotational symmetry of octagon ring, the proposed MRM will be polarization-independent for normally incident THz waves with electric field along x- direction (TE mode) and y- direction (TM mode). The pitch of the unit cell array, p = 120 μm. Dimension of each of the cantilever is 45 μm in length (cl) and 5 μm in width (cw) as shown in Fig. 2(b). The cantilevers are bilayer structures made of 500 nm aluminum (Al) on top of 50 nm aluminum oxide (Al_2_O_3_) dielectric layer, which is fabricated on a silicon (Si) substrate using CMOS compatible process^29^. Silicon-di-oxide (SiO_2_) layer of 100 nm thickness is used as the sacrificial layer. For the release step, vapor hydrofluoric acid (VHF) based dry isotropic etching is used. After the release process, the cantilevers are bent up due to residual stress in the bilayer structure (i.e. Al and Al_2_O_3_). Hence the tip displacement of released cantilevers with respect to Si substrate, “g”, is much higher than the sacrificial layer thickness of 100 nm. The optical microscope (OM) image of the fabricated metamaterial array and scanning electron microscope (SEM) image of the unit cell with eight released cantilevers are shown in Fig. 2(a,b), respectively. Based on the variation of sacrificial etch release time, two devices were fabricated. When the MRM was released for limited release time of 8–10 mins, the tip displacement was measured to be 2.5 μm, and when the release time was increased to 20 mins or higher, the tip displacement saturated at 4.75 μm. The metamaterial devices with limited release time of are termed as “DL500” and that with saturated release time is termed as “DS500”. In order to achieve active tuning, voltage (V_A_) is applied across the released cantilevers and Si substrate.

**Figure 3:**
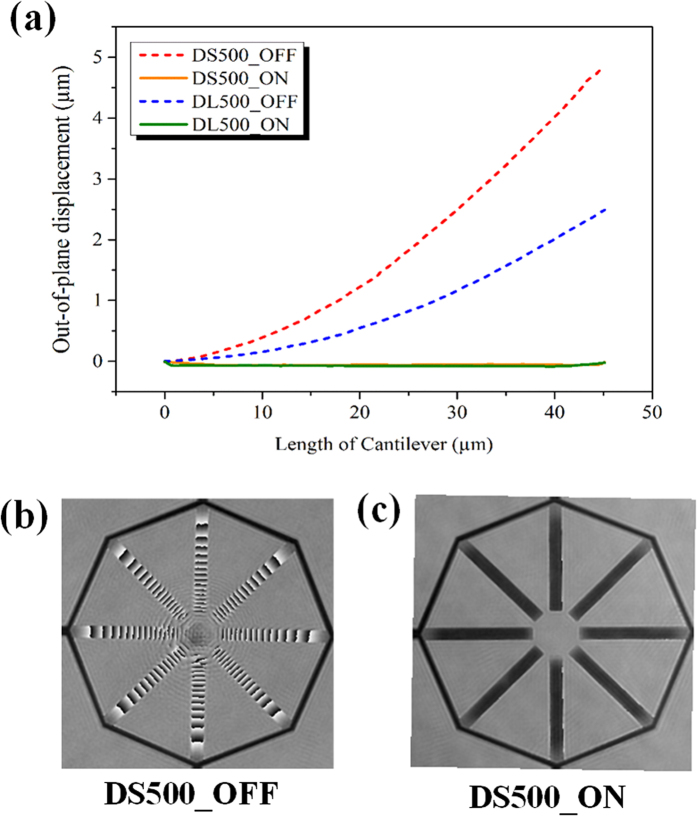
(**a**) Measured single cantilever profile for DS500 and DL500 in OFF and ON states. (**b**) The phase image of released cantilevers for DS500 unit cell in OFF state shows continuously varying phase information due to increasing out-of-plane deformation. (**c**) The phase image of DS500 unit cell in ON state, the phase is uniform throughout the cantilever, indicating that the cantilevers are parallel and in physical contact with Si substrate below.

As V_A_ is increased, the released cantilevers move towards the Si substrate to a position where the attractive electrostatic force balances the restoring force due to cantilever deformation. When V_A_ is greater than the critical pull-in voltage (V_PI_), the electrostatic force will be much higher than the restoring spring force and so the released cantilevers will come in physical contact with Si substrate^41^,^42^,^43^. The initial state when the cantilevers are bent up after release step is termed as “OFF” state. And when all the cantilevers are in contact with the Si substrate under an applied voltage, V_A_ > V_PI_, it is termed as “ON” state. The measured cantilever profiles for DL500 and DS500 at “OFF” and “ON” states are shown in Fig. 3(a). The phase diagram of the DS500 MRM unit cell at “OFF” and “ON” states is shown in Fig. 3(b,c), respectively. When the voltage is turned back to 0 V, the cantilevers will return to the initial OFF state, thereby ensuring repeatable operations for these MRMs.

## Simultaneously Switchable Dual band Polarization-independent characteristics

**Figure 4:**
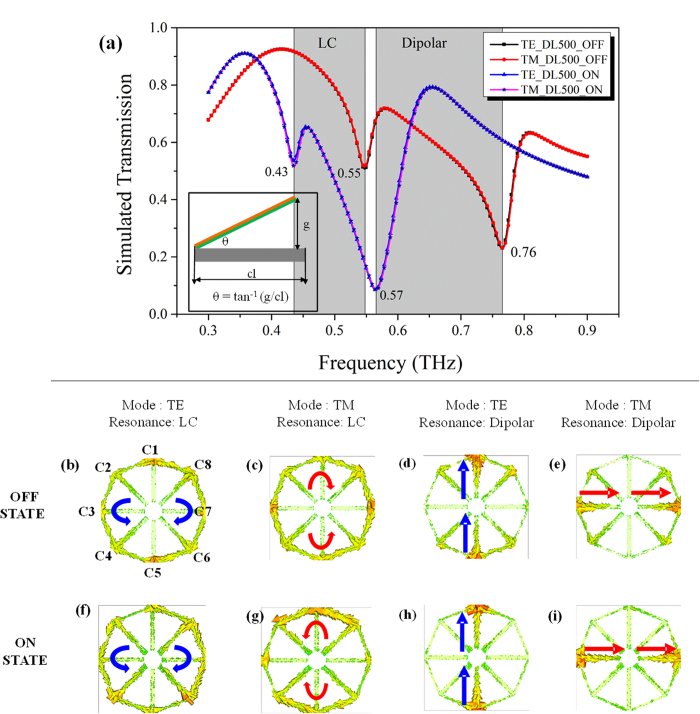
(**a**) Simulated transmission spectra of DL500 in ON and OFF states for TE and TM mode of incidence. (**b**–**i**) The simulated surface current for eLC and dipolar mode resonances for TE and TM in OFF and ON states.

Finite-Difference Time-Domain (FDTD) based EM simulations were performed to explore the resonance mechanisms and to study the effect of resonance frequencies with varying release height of microcantilevers. For the simulations, the DL500 was considered and based on the device dimensions, the ON state resonances were determined to be at 0.43 THz and 0.57 THz and the OFF state resonances were determined to be at 0.55 THz and 0.76 THz, as shown in the simulated transmission spectra in Fig. 4(a). The response of DL500 remains polarization independent for TE and TM modes in both the actuation states. In order to further elucidate the resonance mechanisms, the surface current distributions in the MRM at different resonance frequencies in “ON” and “OFF” states were simulated as shown in Fig. 4(b-i). In case of TE mode resonance at 0.55 THz for DL500_OFF as shown in Fig. 4(b) the circulating current is induced in the cantilevers along the paths C2–C4 and C8-C6 cantilevers and are out of phase with each other. Both these path formed along cantilevers C2–C4 and C8-C6 forms a gap along the X-direction, thereby forming an electrical split ring resonator kind off structure and hence shows the excitation of electrical inductive-capacitive (eLC) mode resonance^44^,^45^,^46^,^47^. In case of TM incidence, the similar circulating current due to eLC resonance is induced in the cantilevers along C2–C8 and C4–C6 at 0.55 THz as shown in Fig. 4(c), that is π/2 rotated compared to the TE case. In case of the higher resonant frequency for TE mode at 0.76 THz for DL500_OFF case, it shows induced current along the cantilevers, C1–C5, which are in phase with each other and also along the same direction as incident electric field (E_X_) as shown in Fig. 4(d). This suggests higher order dipolar mode resonance. Similarly for the TM incidence mode, the current induced in C3–C7 cantilevers is in phase and along E_Y_ as shown in Fig. 4(e). Owing to identical geometry of all eight cantilevers and symmetry of the unit cell with respect to the incident E field, the occurrence of these eLC and dipolar resonances are at the same frequency for both TE and TM mode. Hence, the resonance behavior of the metamaterial remains preserved in both ON and OFF states for TE and TM incident modes.

For DL500 in ON state, the resonance at 0.43 THz shows a circulating current configuration and indicates electrical inductive-capacitive (eLC) mode excitation. While at 0.57 THz, the surface current configuration is along the same direction as incident E field, and hence suggests dipolar mode excitation. Compared to the ON state, the effective capacitance of the metamaterial is decreased in OFF state due to the increased air gap between the released cantilever and Si substrate. Due to this reduced effective capacitance, the OFF state resonance frequencies will be shifted to higher frequencies. The amount by which the OFF state resonance frequency shifts is primarily determined by the initial tip displacement of the released cantilevers^32^.

**Figure 5:**
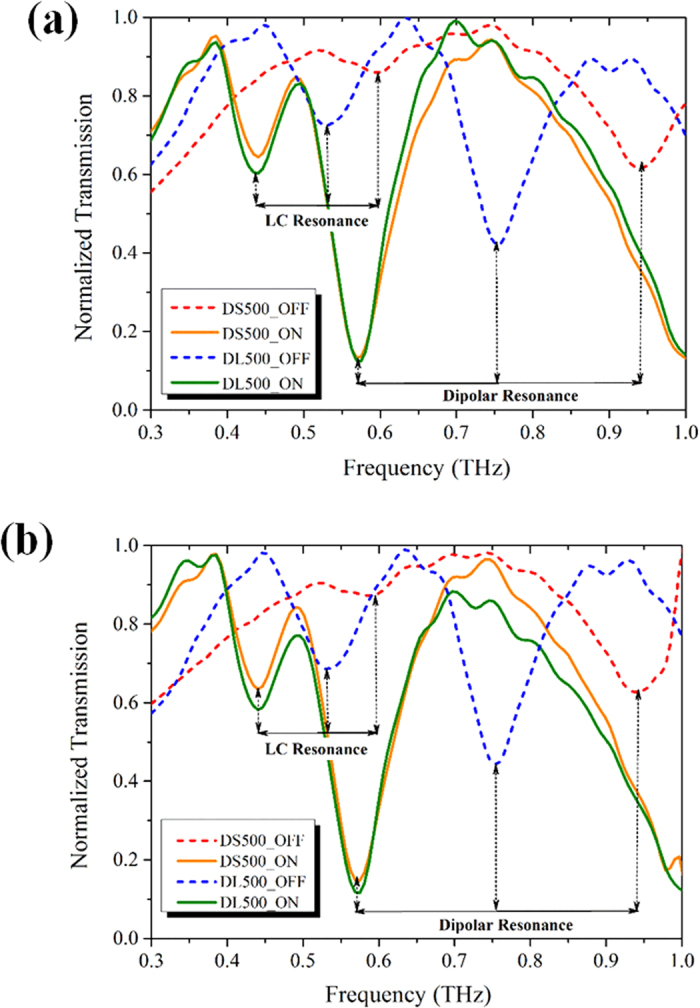
Measured THz transmission spectra for DS500 and DL500 devices in ON and OFF states in (**a**) TE mode and (**b**) TM mode, respectively

Figure 5(a,b) shows the measured TE and TM mode transmission spectra for DL500 and DS500 at normal incidence in both ON and OFF states for the spectral region of 0.3–1 THz, respectively. The ON state eLC and dipolar mode resonances for both devices are at 0.44 THz and 0.57 THz, respectively. In OFF state, the eLC mode resonant frequency for DL500 and DS500 is at 0.53 THz and 0.6 THz, respectively, while the dipolar mode resonance for DL500 and DS500 occurs at 0.75 THz and 0.94 THz, respectively. The tuning range for the eLC and dipolar mode for DL500 is 0.09 THz and 0.18 THz, and for DS500 is 0.16 and 0.37 THz, respectively. Hence, we can observe that DS500 in OFF state has a larger shift compared to DL500, because of the higher tip displacement of released cantilevers for DS500. Both DL500 and DS500 MRMs shows almost identical THz spectral response for TE and TM incidence modes, thereby experimentally confirming the polarization independent charactertics with improved tuning range at eLC and dipolar mode excitations for these devices.

## Geometrical optimization for enhanced electro-optic switching performance

It is also of great interest to note that the OFF state resonance of DS500 is much weaker than the DL500. Hence, with increasing air gap between the released cantilevers and Si substrate, the strength of the resonances of proposed MRMs can be significantly reduced as shown in Fig. 5. If we achieve high enough tip displacement, we can ideally make the OFF state resonance to be immeasurable, i.e. the resonance strength to be as small as the background noise signal. Then the proposed MRMs can operate as an effective modulator not just at a single frequency, but in multiple frequencies simultaneously in the spectral region under consideration. However from Fig. 5a,b, even with initial tip displacement of ~4.75 μm, we can still observe a weak resonance at 0.6 THz in OFF state. In order to further increase the initial tip displacement without changing the ON state resonant frequency, we carried out geometrical optimization study of the cantilevers. The two geometrical parameters, which primarily determine the initial tip displacement after release are the length of the cantilevers and the thickness of Al and Al_2_O_3_ in the bilayer structure. The length of the cantilevers cannot be varied as it defines the ON state resonance frequencies. Also the thickness of Al_2_O_3_ layer cannot be varied significantly as it is deposited using atomic layer deposition (ALD) method. Hence, we experimentally studied the initial tip displacement of released cantilevers with respect to varying Al thickness, while the cantilever length, width and Al_2_O_3_ thickness are fixed. We fabricated three devices with varying Al thickness of 100 nm, 300 nm and 500 nm and these devices are termed as DS100, DS300 and DS500 respectively. As expected, the initial tip displacement of the cantilevers was measured to be approximately 11 μm, 8 μm and 4.75 μm for DS100, DS300 and DS500 MRMs, respectively. The corresponding pull-in voltages for these MRMs were measured as 40 V, 35 V and 30 V, respectively. Even though the initial air gap for thinner Al cantilevers is doubled, the V_PI_ is only slightly changed. This is because of the fact that both the electrostatic force due to an increased air gap and the restoring force due to a lower spring constant for thinner cantilevers are reduced simultaneously. More importantly, the breakdown voltage of Al_2_O_3_ dielectric layer is around 0.7 V/nm; hence, it is important to operate the devices below these voltages^48^. For the reported metamaterial devices, the actual spacing between the suspended Al electrode and Si substrate is formed by 50 nm Al_2_O_3_ and 100 nm air gap, which increases towards the cantilever tip and so the applied voltage of 40 V can be used. This potential limitation can be easily overcome by increasing the Al_2_O_3_ layer thickness.

**Figure 6:**
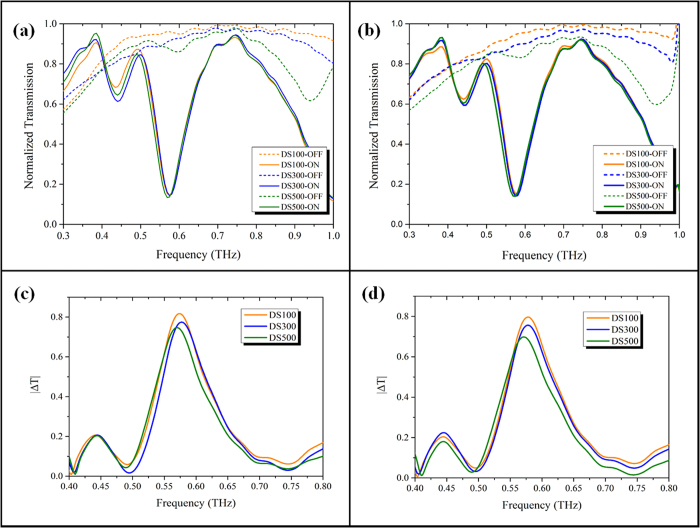
Measured THz transmission spectra for DS100, DS300 and DS500 devices in ON and OFF states in (**a**) TE mode and (**b**) TM mode, respectively. Calculated transmission change between measured ON and OFF state spectra for DS100, DS300 and DS500 devices in (**c**) TE mode and (**d**) TM mode, respectively.

The THz transmission spectra were measured for DS100, DS300 and DS500 in ON and OFF states for TE and TM modes as shown in Fig. 6(a,b), respectively. The ON state resonances for all three devices occur approximately at the designed value of 0.44 THz and 0.57 THz for eLC and dipolar mode, respectively. The slight shift in the ON state resonant frequencies for these devices could be caused due to the variation in Al thickness^48^. However the influence of metal thickness on the ON state resonant frequency is very minimal. For DS500, the weak OFF state eLC and dipolar resonances are observed as shallow dips at 0.6 THz and 0.94 THz, respectively. However, in case of DS300 and DS100, the intensity of eLC resonance is so weak that it cannot be resolved in the measurement and the dipolar resonance is shifted beyond the spectral region under consideration. In the spectral region of 0.3–1 THz, both DS300 and DS100 devices do not show any detectable resonance behavior. The DS100 shows the highest OFF state transmission without any resonances. In order to characterize the performance of the proposed MRM as an electro-optic modulator, we calculated the absolute change in measured transmission between the ON and OFF states as |ΔT(ω)| = |T_OFF_(ω) − T_ON_(ω)|. Figure 6(c,d) shows the |ΔT(ω)| for all these MRMs at TE and TM incidence modes, respectively. The absolute change in measured transmission at 0.44 THz for eLC mode resonance is approximately 0.2. The maximum change in transmission occurs at the ON state dipolar resonance at 0.57 THz for all devices. The |ΔT(ω)| at 0.57 THz is increased from 0.7 to 0.8 with respect to DS500 and DS100 for both TE and TM modes. The value of |ΔT(ω)| can be increased to close to its ideal value of 1, by reducing the Al thickness to achieve higher transmission in OFF state and increasing the ON state resonance strength to almost zero. However, the skin depth at THz spectral region will limit the further reduction in thickness of Al layer. Below this limit, the ON state resonance will start to get weaker, thereby adversely affecting the |ΔT(ω)| value.

The geometrical thickness optimization proposed here to improve the modulation depth, can also be adopted to achieve the OFF state resonance at any desired frequency of interest. This method can be precisely controlled and is more effective than the aforementioned approach of controlling the sacrificial layer release time. The proposed MRMs are polarization insensitive for both TE and TM modes due to the π/2 rotational symmetry of the unit cell. These MRMs with multiple operational cycles are fabricated using CMOS compatible process on an 8” Si wafer. Ideally, these MRMs can also be operated in AC mode to achieve high speed electro-optic modulators.

## Prospective of multiband polarization-independent terahertz MEMS metamaterials

Arrays of these MRMs can also be used to realize spatial light modulators (SLMs) by individually controlling each of the pixels forming the SLM^14^,^15^. These SLMs can then enable compressive THz imaging using a single detector element^49^. These MRMs can also be further used in the absorber configuration by adding in an optimized thickness of dielectric layer and bottom reflective layer. This can enable tunable THz absorbers, which are critical for multicolor imaging in THz region using a single device without the need for tunable filters^50^,^51^,^52^. Since the microactuators in the MRMs can respond to different external forces, they can also be used in various types of sensing applications such as flow sensors^53^, pressure sensors, temperature sensor, chemical sensor^53^, biomolecule sensor, etc. Additionally these terahertz electro-optic devices and sensors will enable system level applications such as miniaturized terahertz spectroscopy, medical imaging^54^,^55^, security surveillance^56^, next generation high speed wireless communication^57^ in the near future.

In summary, we experimentally demonstrate a THz MEMS reconfigurable metamaterial with polarization insensitive charactertics at eLC and dipolar mode resonances for TE and TM incidence modes. The proposed isotropic MRM has a large switching range of 0.37 THz and as a modulator with a sharp transmission change of 0.7 ~ 0.8 at 0.57 THz. We have proposed and demonstrated a unique design methodology for improving the electro-optic performance, through simple geometrical optimization technique and is also highly scalable to any EM spectral region. The proposed MRMs were fabricated using a CMOS compatible process and can be readily incorporated in batch manufacturing lines. Hence the proposed MRM with isotropic characteristics, improved switching performance, repeatable operations, versatile design variation and high yield fabrication process is an ideal candidate to be the enabling technology for the realization of numerous exciting terahertz applications in the near future.

## Methods

### Electromagnetic Simulation

Finite-difference time-domain (FDTD) numerical simulations were conducted to calculate the transmission spectra and the electromagnetic field and surface current distributions corresponding to the resonance modes with normally incident THz waves of either TE or TM polarization. Full-field electromagnetic wave simulations were performed using the commercial simulation software Computer Software Technology (CST) Microwave studio 2009. For the material property, Aluminum was modelled as lossy metal with conductivity of 1e7 S/m. Aluminum oxide and Silicon were modelled as lossless dielectric with dielectric constant of 9.5 and 11.9, respectively. In the simulation, a single unit cell of the metamaterial structures was simulated with unit cell boundary conditions employed in axial directions orthogonal to the incident waves. The perfectly matched layers are applied along the propagation of the electromagnetic waves. Plane waves were incident into the unit cell from the port on the metal side, while the transmission spectrum was determined from the port placed at the other side of metamaterial. In the meanwhile, field monitors are used to collect the electric fields, magnetic fields and the respective surface currents at different resonance frequencies.

### Fabrication

Firstly an 8 inch p-doped silicon wafer with a resistivity of 3 Ω cm is rinsed with DI water. Blanket low-pressure chemical vapor deposition (LPCVD) of a 100-nm-thick silicon oxide (SiO_2_) is carried out on the Si wafer. The SiO_2_ acts as the sacrificial layer. First lithography step is used to define and etch the SiO_2_ layer with reactive ion etching to form the anchor region of the metamaterial. Then, a 50-nm-thick aluminum oxide (Al_2_O_3_) layer is deposited using atomic layer deposition process. Now the Al_2_O_3_ will be in contact with Si substrate at the anchor region and at the other region will be on top of sacrificial SiO_2_ layer. Following it, a 500-nm-thick aluminum (Al) layer is sputtered. Then, the Al/Al_2_O_3_ bilayer is patterned with the second mask for the metamaterial pattern. Finally, vapor HF is used to isotropically etch the sacrificial SiO_2_ layer. As the anchor region of the metamaterial is fixed directly to the Si substrate, isotropic VHF etch is carried out until all the SiO_2_ is removed without the need for time control^32^. Alternatively, stress beam structures can be fabricated using a single mask process with time controlled release step^58^,^59^. Finally when the VHF etching process is completed, the bilayer cantilevers are suspended over the Si substrate with air gap between them.

### Electromechnical Characterization

The deflection profile of released cantilevers for DL500 and DS500 were measured using reflection digital holographic microscope (R-DHM). The released chips are wire bonded to a PCB. Then a voltage power supply is used to bias the released device. Si substrate is kept at ground potential, and the cantilevers are positively biased. The voltage is increased between the cantilevers and Si substrate and when the applied voltage is higher than the pull in voltage, the cantilevers will come in physical contact with the Si substrate. The Al_2_O_3_ layer beneath the Al layer, will ensure that there is no current flowing from Al layer to Si substrate, when pull in occurs. This is crucial because if the current flows then the temperature will raise up locally at the Al/Si junction, thereby melting the Al tips with the Si substrate, and hence permanently damaging the device. At different applied voltage, the cantilever profile can be measured directly from the R-DHM unit.

### Terahertz Transmission Measurement

The THz transmission spectra characterization were measured using Teraview 3000 time-domain spectroscopy (THz-TDS) system. The THz waves were incident normally with electric field along X- direction (TE mode) to the sample placed in the nitrogen filled chamber. The wire bonded chip was then applied with desired voltage from an externally connected Agilent E3646A power supply. Depending upon the voltage applied, the position of the cantilevers can be controlled. The transmission spectra were then measured for the MEMS metamaterial in both ON and OFF states. For measuring the TM mode response, the chip was rotated by 90° and the experiments were repeated. The transmission spectra were normalized with respect to the transmission of the pure silicon substrate of same thickness as the samples.

## Additional Information

**How to cite this article**: Pitchappa, P. *et al.* Microelectromechanically tunable multiband metamaterial with preserved isotropy. *Sci. Rep.* **5**, 11678; doi: 10.1038/srep11678 (2015).
